# Diagnostic Performance of Kaiser Score for Characterization of Breast Lesions on Modified Abbreviated Breast MRI and Comparison with Full-Protocol Breast MRI

**DOI:** 10.3390/jcm14010264

**Published:** 2025-01-05

**Authors:** Merve Erkan, Seray Gizem Gur Ozcan

**Affiliations:** 1 Department of Radiology, Bursa City Hospital, 16110 Bursa, Turkey; 2 Department of Radiology, Bursa Yuksek Ihtisas Training and Research Hospital, 16310 Bursa, Turkey; seraygizemgur@yahoo.com.tr

**Keywords:** Kaiser score, abbreviated breast MRI, full-protocol MRI, breast cancer

## Abstract

**Background**: This study aimed to evaluate the diagnostic performance of the Kaiser score (KS) on the modified abbreviated breast magnetic resonance imaging (AB-MRI) protocol for characterizing breast lesions by comparing it with full-protocol MRI (FP-MRI), using the histological data as the reference standard. **Methods**: Breast MRIs detecting histologically verified contrast-enhancing breast lesions were evaluated retrospectively. A modified AB-MRI protocol was created from the standard FP-MRI, which comprised axial fat-suppressed T2-weighted imaging (T2WI), pre-contrast T1-weighted imaging (T1WI), and first, second, and fourth post-contrast phases. Two radiologists reviewed both protocols, recording the KS for each detected lesion. Sensitivity, specificity, and positive and negative predictive values, as well as accuracy, were calculated for each protocol. Receiver operating characteristic (ROC) analysis was performed to determine the diagnostic performance of the modified AB-MRI compared to the FP-MRI. **Results**: In total, 154 patients with 158 histopathologically proven lesions (107 malignant, 51 benign) were included. For the diagnostic performance of the KS for modified AB-MRI and FP-MRI, the sensitivity was 96.3% vs. 98.1%, the specificity was 78.4% vs. 74.5%, PPV was 90.4% vs. 89%, NPV was 90.9% vs. 95%, and the diagnostic accuracy was 90.5% vs. 90.5%. The area under the curve (AUC) obtained from the ROC curve analysis was 0.873 and 0.863 for modified AB-MRI and FP-MRI for reader 1, respectively, and 0.859 and 0.878 for modified AB-MRI and FP-MRI for reader 2, respectively, (*p* < 0.001). **Conclusions**: Our modified AB-MRI protocol revealed comparable results in terms of the diagnostic value of the KS in characterizing breast lesions compared to FP-MRI and reduced both scanning and interpretation time.

## 1. Introduction

Breast cancer is the most common cause of malignancy in women worldwide and is considered one of the leading causes of cancer-related mortality and morbidity [1]. Early and accurate diagnosis and timely treatment can improve the prognosis of patients with breast cancer, reduce mortality, and increase cure rates [2]. Breast magnetic resonance imaging (MRI) is the most sensitive imaging modality for detecting breast cancer and is superior to both mammography and ultrasonography [3,4,5]. Therefore, indications for breast MRI have increased over the past decade, including screening high-risk women, problem solving, preoperative staging, evaluating the response to neoadjuvant chemotherapy, and nipple discharge [6,7]. However, the long acquisition time (approximately 35 to 40 min) required for full-protocol MRI (FP-MRI) increases costs, prolongs the interpretation time, and may also cause patient discomfort. To overcome these limitations, the abbreviated breast MRI (AB-MRI) protocol has been introduced in screening and diagnostic settings [8]. This protocol demonstrates diagnostic accuracy and cancer detection rates comparable to those of conventional FP-MRI [9].

The American College of Radiology (ACR) Breast Imaging Reporting and Data System (BI-RADS) is widely used in the interpretation of breast lesions. It provides uniform terminology and a standardized classification for breast lesions [10]. However, the accurate diagnosis of breast diseases via ACR BI-RADS may require considerable experience, so it is challenging for young radiologists. For this purpose, the Kaiser scoring system, an evidence-based clinical decision-making tool for the characterization of breast lesions, was developed for breast MRI. The Kaiser score (KS) has a classification tree flowchart structure that includes five major diagnostic BI-RADS lexicon criteria (root sign [presence of spiculations], a signal intensity–time curve type, lesion margins, internal enhancement, and the presence of edema). The resulting score reflects the increased likelihood of malignancy, ranging from 1 to 11 [11]. Applying the KS in breast MRI interpretation has improved diagnostic accuracy and inter-reader agreement, especially for less experienced radiologists. In systematically evaluating lesions with a structured scoring system, the KS can reduce diagnostic uncertainty. This approach may help distinguish benign from malignant lesions more effectively, potentially reducing the number of unnecessary biopsies [12].

One of the most important diagnostic criteria included in the flowchart is the signal-intensity (SI)–time curve type of dynamic contrast enhancement. The shape of the SI–time curve is determined by initial and delayed enhancement [11]. The most commonly described AB-MRI protocol consists of the most essential sequences, including pre-contrast T1-weighted imaging (T1WI) and a single post-contrast T1WI scan [8]. However, this protocol also has several inherent challenges, such as high recall and false positive rates due to the absence of delayed contrast-enhanced images and fat-suppressed T2-weighted imaging (T2WI). To evaluate the ability of the KS to characterize breast lesions, we created a modified AB-MRI protocol by adding T2WI and delayed-phase post-contrast T1WI to the classical AB-MRI protocol.

Our study aimed to evaluate the diagnostic performance of the KS on modified AB-MRI for characterizing breast lesions by comparing it with that of FP-MRI, using histological data as the reference standard.

## 2. Materials and Methods

### 2.1. Study Design

This was a single-center retrospective study involving women who underwent breast MRI between January 2020 and December 2023 at our department for the evaluation of suspicious findings on mammography or ultrasonography. Only patients with MRI enhanced lesions and histopathological correlations were included. The exclusion criteria were as follows: the absence of detectable breast lesions on MRI, suspected lesions on MRI without pathologic confirmation or studies significantly affected by motion artifacts. This retrospective study was approved by the institutional board, and written informed consent was waived because of the retrospective nature of the investigation.

### 2.2. MRI Equipment and Technique

All MRI examinations were performed with a 1.5 Tesla MRI scanner (Optima 360^®^, GE Healthcare, Waukesha, WI, USA) using a dedicated 12-channel breast coil while the patient was in the prone position. All women underwent FP-MRI examination on the same MRI machine and with the same protocol. Gadolinium contrast material was administered intravenously, with 0.1 mmol/kg as a bolus injection with an injector followed by 15 mL of saline solution.

The standard FP-MRI consisted of axial fat-suppressed T2WI, axial T1WI, sagittal fat-suppressed T2WI, diffusion weighted imaging (DWI) with apparent diffusion coefficient (ADC) maps, axial fat-suppressed pre-contrast T1WI, and axial dynamic contrast enhanced series acquired five times after contrast agent injection. Additionally, subtraction and maximum intensity projection (MIP) images were generated automatically. The total acquisition time for standard FP-MRI was approximately 35–40 min. The modified AB-MRI protocol was created from sequences obtained from standard FP-MRI. This data set comprised axial fat-suppressed T2WI, pre-contrast T1WI, and first, second, and fourth post-contrast phases. The total acquisition time for the modified AB-MRI was 12–14 min. The subtraction and MIP images were generated automatically and did not require additional scan time.

### 2.3. Image Analysis and Interpretation

Both the FP-MRI and modified AB-MRI examinations were assessed retrospectively by two radiologists (Reader 1 with 10 years of diagnostic experience, and Reader 2 with 8 years of diagnostic experience). Radiologists were blinded to the patients’ clinical history, prior imaging studies and histopathological results. The suspicious lesion with a histopathological result was noted in advance so that each radiologist analyzed the same lesion. If any conflict occurred between the two observers’ interpretations, the final decision was reached by consensus.

MR images were retrieved from the Picture Archiving and Communication System (PACS). FP-MRI includes all available sequences described above. Modified AB-MRIs were created from FP-MRIs and saved as anonymized Digital Imaging and Communications in Medicine (DICOM) files by a technician. Each reader first read the modified abbreviated protocol and then read the full protocol approximately one-month later to minimize the memory bias.

**Figure 1:**
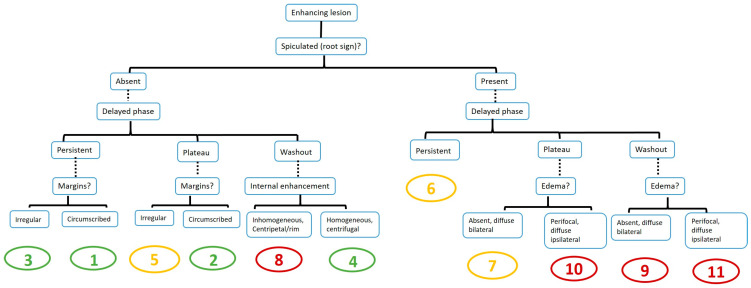
The tree flowchart of the Kaiser score [11]. The resulting score is associated with an increasing risk of malignancy (from 1 to 11).

**Figure 2:**
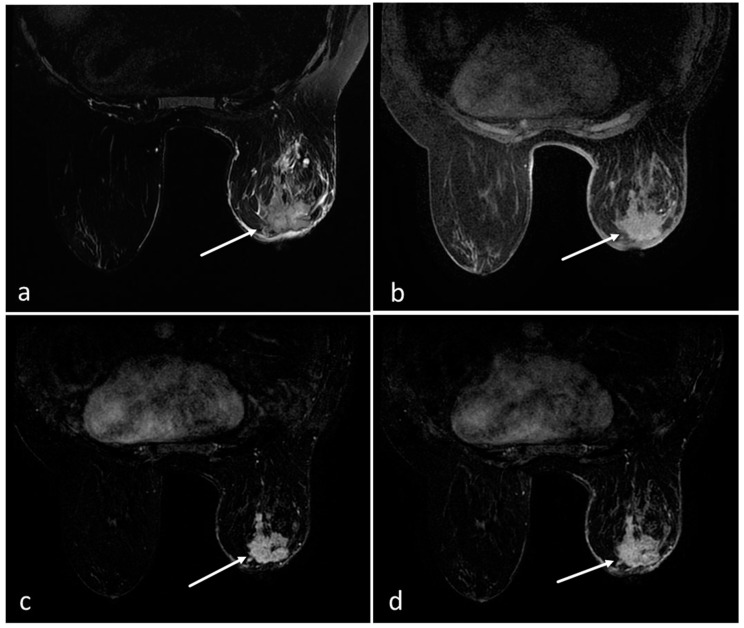
Axial T2 weighted (**a**), axial pre-contrast T1 weighted (**b**), dynamic post-contrast first-phase subtraction (**c**), and dynamic post-contrast fourth-phase subtraction, (**d**) sequences from the modified abbreviated breast MRI demonstrating a 41 mm spiculated mass lesion (arrows) in the retroareolar area of the right breast with post-contrast washout and perilesional edema on T2 weighted image (Kaiser score = 11). Histopathology revealed invasive carcinoma.

Age, menopausal status, amount of fibroglandular tissue (FGT), background parenchymal enhancement (BPE), lesion size, and lesion localization were recorded. The lesions were divided into mass and non-mass enhancement (NME). The size of the enhancing lesion was defined as the largest diameter. The readers were asked to classify all enhancing lesions using the KS as described in the literature [11]. This score combines five major diagnostic BI-RADS lexicon criteria (root sign [presence of spiculations], internal enhancement, lesion margins, SI–time curve type, and the presence of edema) in a flowchart-like algorithm (Figure 1) [11]. If there was an enhanced lesion, the presence of spiculation, kinetic curve type, lesion margin characteristics, internal enhancement pattern, and the presence of edema were evaluated to calculate the KS. The resulting score reflects the increased likelihood of malignancy, ranging from 1 to 11 (Figure 2). Scores greater than 4 require biopsy. A diagnostic category was assigned to each lesion that was biopsied. Interpretation times were recorded for the modified AB-MRI and FP-MRI for both readers.

### 2.4. Statistical Analysis

All statistical analyses were carried out using the SPSS 23 statistical software (SPSS Inc., Chicago, IL, USA). The sensitivity, specificity, positive predictive values (PPVs), negative predictive values (NPVs), and 95% confidence intervals (CIs) of the KS in FP-MRI and modified AB-MRI for the characterization of breast lesions were calculated. The diagnostic performance of the Kaiser score was interpreted using receiver operating characteristic (ROC) analysis, and the area under the curve (AUC) was calculated. A Kaiser score of 4 was accepted as the cutoff value. The intra-class correlation coefficient (ICC) was used to match the numerical Kaiser scores between the two readers. Interrater agreement between the two readers was analyzed using Cohen’s kappa coefficient. The values (κ) were interpreted as follows: poor (κ is less than 0.20), fair (κ ranges from 0.21 to 0.40), moderate (κ ranges from 0.41 to 0.60), good (κ ranges from 0.61 to 0.80), and excellent (κ is greater than 0.81) [13]. A *p*-value of less than 0.05 was considered statistically significant.

## 3. Results

A total of 154 patients with 158 histopathologically proven lesions were included in this study. The final histopathology revealed that 67.7% (n = 107) of the lesions were malignant and that 32.3% (n = 51) were benign. The detailed histopathological diagnoses and subtypes are given in Table 1. The mean age of the patients with malignant lesions was 55.2 ± 12.6 years and that of the patients with benign lesions was 38.8 ± 12.8 years. The difference between groups in terms of mean age was statistically significant (*p* < 0.001).

A total of 143 masses (90.5%) and 15 (9.5%) NME cases were diagnosed. The mean ± standard deviation of the size of the detected mass lesions was 28.3 ± 16.9 mm, and that of the NME was 54.0 ± 23.5 mm. Table 2 shows the patient characteristics and MRI findings of 158 histopathologically proven benign and malignant lesions.

**Figure 3:**
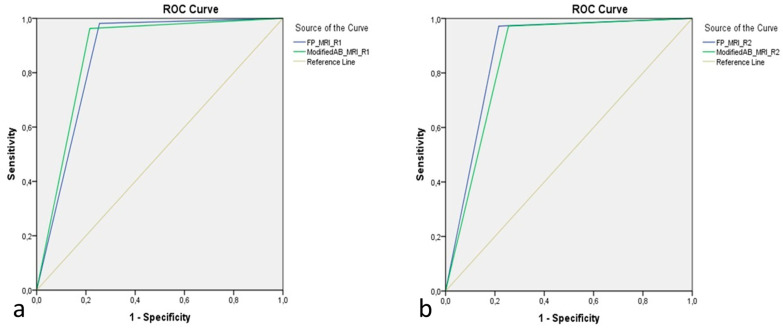
Comparison of diagnostic performance of the Kaiser score between modified AB-MRI and FP-MRI according to the reader. The area under the curve according to the receiver operating characteristic analysis is 0.873 for the modified AB-MRI vs. 0.863 for the FP-MRI for reader 1 (**a**) and 0.859 for the modified AB-MRI vs. 0.878 for the FP-MRI for reader 2 (**b**) (*p* < 0.001).

The diagnostic performance of the KS for differentiating between benign and malignant lesions via modified AB-MRI and FP-MRI was as follows: sensitivity, 96.3% vs. 98.1%; specificity, 78.4% vs. 74.5%; PPV, 90.4% vs. 89%; NPV, 90.9% vs. 95%; and diagnostic accuracy, 90.5% vs. 90.5%, respectively (Table 3). The AUCs obtained from the ROC curve analysis were 0.873 and 0.863 for modified AB-MRI and FP-MRI, respectively, for reader 1 and 0.859 and 0.878 for modified AB-MRI and FP-MRI, respectively, for reader 2 (*p* < 0.001) (Figure 3). For both protocols, the cutoff point for optimally classifying patients was KS > 4.

With modified AB-MRI, the number of false positive cases was 11, represented by suppurative inflammation, granulomatous mastitis, and cholesterol granuloma, which showed irregular margins and marked inhomogeneous enhancement that mimicked malignancies. Four false negative cases presented with high T2 signals and persistent enhancement that mimicked benign lesions and were invasive lobular carcinomas.

In the evaluation of the agreement between readers in terms of KS evaluation in modified AB-MRI and FP-MRI, the interobserver ICCs were 0.969 (0.957–0.977) and 0.965 (0.952–0.974), respectively, which was considered excellent agreement. The mean interpretation time was 2.2 min for modified AB-MRI and 5.7 min for FP-MRI. The difference was statistically significant, with *p* < 0.001.

## 4. Discussion

This study revealed that modified AB-MRI has diagnostic accuracy comparable to that of FP-MRI for the KS in characterizing breast lesions. In addition, we demonstrated reduction in the total acquisition and interpretation time with near-perfect inter-observer agreement.

Breast MRI is the most sensitive method for breast cancer detection and is currently recommended as a supplemental screening tool for women at high risk for breast cancer [14,15,16]. Additionally, MRI is widely used for problem solving, preoperative staging, the evaluation of the response to neoadjuvant chemotherapy, and nipple discharge [6,7]. Despite these recommendations, the use of breast MRI is restricted due to the long scanning time of a full protocol, which increases patient anxiety and discomfort, and high costs. Abbreviated MRI protocols that shorten the scanning time by reducing the number of sequences. The most commonly acquired AB- MRI protocol consists of one pre-contrast sequence and one early-phase (first) post-contrast sequence, which lacks a delayed-phase post-contrast sequence [8]. Therefore, it does not allow for the assessment of the enhancement pattern or analysis of time–intensity curves to characterize lesions. Benign lesions usually present with slow or delayed initial enhancement, whereas initial rapid enhancement and rapid washout are highly indicative of a malignant mass. However, AB-MRI without delayed-phase post-contrast sequences may misinterpret slowly enhancing cancers such as invasive lobular carcinomas or low-grade ductal carcinoma in situ [17]. Therefore, due to the lack of delayed post-contrast images, lesions may be difficult to interpret or identify. In some studies, T2WI has been included in the AB-MRI protocol [18,19,20]. T2WI may be helpful in lesion analysis; in some cases, T2 hyperintensity may enable a benign finding. Mann et al. reported that T2WI is very helpful in lesion characterization [20]. Our modified AB-MRI protocol differs from the classic AB-MRI protocol in that both delayed-phase post-contrast sequences and T2WI are required. In this state, our modified AB-MRI protocol serves as a full-protocol breast MRI without losing diagnostic accuracy, and there is a need to bring patients for further MRI evaluation, which can be acquired in approximately 12–14 min. Furthermore, although Grimm et al. and Lee et al. previously described a similar abbreviated protocol, we are the first to evaluate the diagnostic performance of the KS on modified AB-MRI for characterizing breast lesions by comparing it with that of FP-MRI, using histological data as the reference standard [21,22].

Our study supports the literature, showing that modified AB-MRI is sensitive for characterizing breast lesions, with less scanning and interpretation time. The sensitivity of modified AB-MRI was as high as that of FP-MRI (96.3% vs. 98.1%). The diagnostic accuracies of the two protocols were equal (90.5% vs. 90.5%). Furthermore, the specificity and PPV of modified AB-MRI were slightly higher than those of FP-MRI (78.4 vs. 74.5% and 90.4 vs. 89%, respectively), which indicated that false-positive findings were less frequent with modified AB-MRI. Lee et al. found that, compared with classic AB-MRI, modified AB-MRI with additional T2WI and delayed-phase post-contrast T1WI scans tended to show higher average sensitivity, comparable average specificity, and a higher average AUC [22]. Heacock et al. evaluated the use of an abbreviated MRI protocol with T2WI and found that abbreviated MRI has a high rate of detection for known breast cancer and a short interpretation time [18]. Grimm et al. compared two AB-MRI protocols for breast cancer detection, including T2WI (abbreviated 1) and second-pass T1-weighted post-contrast (abbreviated 2), with a standard protocol and reported no significant difference in sensitivity between the abbreviated 1 (86%, *p* = 0.22) or abbreviated 2 (89%, *p* = 0.38) protocols and the full protocol (95%) [21]. Romeo et al. emphasized the usefulness of late post-contrast series in the characterization of breast lesions and their comparability with the standard full diagnostic protocol [23]. Moschetta et al. concluded that an abbreviated combined MRI protocol has the same diagnostic potential as the standard protocol in patients undergoing breast MRI for screening, problem solving, or preoperative staging and is a time-saving method [19]. These studies indicate that although AB-MRI with second or subsequent post-contrast images has comparable performance to FP-MRI, AB-MRI with a single first post-contrast sequence tends to have lower sensitivity for lesion characterization than FP-MRI.

Li et al. demonstrated that contrast agent injection can improve the signal-to-noise ratio and contrast-to-noise ratio of fat-saturated T2 images, thus providing higher quality images for breast lesion characterization [24]. The modified AB-MRI used by Lee et al. involves T2WI being placed between early- and delayed-phase post-contrast sequences to simultaneously shorten the scanning time and benefit from delayed-phase and T2WI scans [22]. In considering these studies, the time period for modified AB-MRI may be further shortened if T2WI is interpolated between early- and delayed-phase post-contrast sequences.

The KS, which provides greater clinical guidance, is a three-step flowchart based on morphological and dynamically relevant features. The resulting score reflects the increased likelihood of malignancy, ranging from 1 to 11 [11]. A KS of 4, which is the currently established cutoff value for biopsy recommendation, was valid for our study group [11,12,25]. In the literature, Woitek et al. reported that the sensitivity and specificity of the KS for differentiating between benign and malignant lesions were 80.6% and 82.5%, respectively, and the AUC was 0.873 [25]. Wang et al. reported a high diagnostic accuracy of the Kaiser score, with an AUC of 0.958 [12]. Milos et al. calculated AUCs of all lesions for three readers to be between 0.865 and 0.902, with sensitivities between 92.7 and 97.6% [26]. For the KS, the authors recommend a visual assessment of kinetic curve types as they consider that it allows for faster evaluation and is less affected by motion and partial volume artifacts [27]. Therefore, in this study, we aimed to investigate the diagnostic performance of the KS in an abbreviated MRI protocol. In our study, the diagnostic accuracy and AUC of the KS for modified AB-MRI were calculated as 90.5% and 0.859–0.873, respectively, which supports the literature. These findings indicate that KS has good practical potential for use in modified AB-MRI.

The mean image acquisition and interpretation times were 35–40 min and 5.7 min, respectively, for the FP-MRI and 12–14 min and 2.2 min, respectively, for the modified AB-MRI, with a statistically significant difference between the two protocols. Additionally, there were no significant differences in diagnostic accuracy between the two protocols. Our total acquisition and interpretation time for modified AB-MRI was longer than that of Kuhl’s acquisition and interpretation time because of the time required to acquire T2WI and post-contrast delayed sequences [8].

Our study has several limitations. First, it was a retrospective study conducted at a single center. Second, the modified AB-MRI protocol was created by extracting relevant images from the FP-MRI and then interpreting them in a separate session due to the retrospective nature of the study. Additionally, one of the greatest challenges in the use of abbreviated MR images is the lack of standardization. Therefore, it is conceivable that future studies should prospectively investigate modified AB-MRI.

## 5. Conclusions

Compared with FP-MRI, our modified AB-MRI protocol revealed comparable results in terms of the diagnostic value of the KS in characterizing breast lesions and reduced both the scanning and interpretation times. This is also a way to expand the use of MRI in breast imaging by improving patient comfort and allowing a greater number of examinations. However, before the widespread use of this modified AB-MRI protocol, it needs to be confirmed in a larger study population including prospective studies.

## Figures and Tables

**Table 1:** Histopathological features of 158 lesions.

Histopathologic Features	n (%)
Benign lesions	51 (32.3)
Fibrocystic change	2 (1.3)
Fibroadenoma	34 (21.5)
Fibrosis-adenosis	2 (1.3)
Granulomatous mastitis	3 (1.9)
Intraductal papilloma	2 (1.3)
Cholesterol granuloma	1 (0.6)
Chronic inflammation	2 (1.3)
Suppurative inflammation	3 (1.9)
Nonspecific inflammation	2 (1.3)
Malign lesions	107 (67.7)
Invasive ductal carcinoma	87 (55.1)
Invasive lobular carcinoma	7 (4.4)
Invasive mixt carcinoma	2 (1.3)
Ductal carcinoma in situ	2 (1.3)
B-cell non-Hodgkin’s lymphoma	1 (0.6)
Apocrine adenocarcinoma	1 (0.6)
Tubular carcinoma	1 (0.6)
Encapsulated papillary carcinoma	1 (0.6)
Solid papillary carcinoma	1 (0.6)
Metaplastic carcinoma	1 (0.6)

**Table 2:** Patient characteristics and MRI features of benign and malignant lesions.

Characteristics	Benign Lesions (n = 51)	Malign Lesions (n = 107)	*p* Value
**Patient characteristics**			
Mean age (range) (years)	38.8 (19–73)	55.2 (24–82)	<0.001
Menstrual status			<0.001
Premenopausal	43 (52.4%)	39 (47.6%)	
Postmenopausal	8 (10.5%)	68 (89.5)	
**MRI features**			
Amount of FGT			<0.001
Fat	2 (66.7%)	1 (33.3%)	
Scattered	5 (10.4%)	43 (89.6%)	
Heterogenous dense	17 (29.8%)	40 (70.2%)	
Extremely dense	27 (54%)	23 (46%)	
BPE			0.567
Minimal	19 (31.7%)	41 (68.3%)	
Mild	17 (28.3%)	43 (71.7%)	
Moderate	10 (35.7%)	18 (64.3%)	
Severe	5 (50%)	5 (50%)	
Lesion size (range) (mm)	24.7 (7–107)	33.6 (8–91)	<0.001
Lesion type			0.565
Mass	45 (31.5%)	98 (68.5%)	
NME	6 (40%)	9 (60%)	
T2 signal intensity of lesion			0.594
High	29 (34.1%)	56 (65.9%)	
Not high	22 (30.1%)	51 (69.9%)	
Root sign (spiculated)			<0.001
Yes	3 (3.2%)	90 (96.8%)	
No	48 (73.8%)	17 (26.2%)	
Margins			<0.001
Circumscribed	37 (97.4%)	1 (2.6%)	
Irregular	11 (40.7%)	16 (59.3%)	
TIC			<0.001
Persistent	30 (85.7%)	5 (14.3%)	
Plateau	16 (21.9%)	57 (78.1%)	
Washout	5 (10%)	45 (90%)	
Internal enhancement			<0.001
Homogenous, centrifugal	33 (62.3%)	20 (37.7%)	
Heterogenous, centripedal/rim	18 (17.1%)	87 (82.9%)	
Edema			<0.001
Yes	37 (45.7%)	44 (54.3%)	
No	14 (18.2%)	63 (81.8%)	

MRI: magnetic resonance imaging, FGT: fibroglandular tissue, BPE: background parenchymal enhancement, NME: non-mass enhancement, TIC: time–intensity curve.

**Table 3:** Sensitivity, specificity, PPV, NPV, diagnostic accuracy, and 95% confidence intervals (in parentheses) of Kaiser score in modified AB-MRI and FP-MRI for characterization of breast lesions.

	Sensitivity (%)	Specificity (%)	PPV (%)	NPV (%)	Diagnostic Accuracy (%)
Modified AB-MRI	96.3 (90.7–98.9)	78.4 (64.6–88.7)	90.4 (84.7–94.1)	90.9 (79.1–96.3)	90.5 (84.8–94.5)
FP-MRI	98.1 (93.4–99.7)	74.5 (60.3–85.7)	89 (83.4–92.8)	95 (82.7–98.7)	90.5 (84.8–94.5)

PPV: positive predictive value, NPV: negative predictive value, AB-MRI: abbreviated breast magnetic resonance imaging, FP-MRI: full-protocol magnetic resonance imaging.

## Data Availability

The data presented in this study can be made available from the corresponding author upon reasonable request. The data are not publicly available due to restrictions pertaining to data privacy.
